# Surgical Treatment for Radiculopathy Due to Spinal Metastasis: A Report of Three Cases

**DOI:** 10.7759/cureus.17762

**Published:** 2021-09-06

**Authors:** Takaki Kitamura, Satoshi Maki, Takeo Furuya, Yasuhiro Shiga, Seiji Ohtori

**Affiliations:** 1 Department of Orthopaedic Surgery, Graduate School of Medicine, Chiba University, Chiba, JPN

**Keywords:** radiculopathy, spinal metastasis, cervical spine, lumbar spine, metastatic tumor

## Abstract

Nonsurgical treatment is the first option in patients with radiculopathy due to spinal metastasis. However, we have to consider surgical management for patients who are resistant to conservative treatment. There are few reports of surgical treatment for radiculopathy due to metastatic spine tumors. We present cases in three patients who underwent surgery for radiculopathy due to spinal metastasis. Case 1 was in an 82-year-old woman with lumbar foraminal stenosis at L5-S1 due to breast cancer metastasis to the right L5-S1 intervertebral foramen. She underwent subtotal tumor resection and posterior lumbosacral decompression and fusion. After the surgery, she was able to walk without pain. Case 2 was in a 70-year-old woman with C8 radiculopathy and amyotrophy due to breast cancer metastasis to the right C7-T1 intervertebral foramen. She underwent anterior cervical decompression and fixation from C6 to T1. After the surgery, the pain in her left upper limb was relieved, but the muscle weakness of her left finger extension remained. Case 3 was in a 72-year-old woman with C8 radiculopathy and amyotrophy due to rectal cancer metastasis to the right side of the C7 vertebral body and pedicle. She underwent tumor resection and left C7-T1 facetectomy. Muscle weakness of her right finger extension and pain improved postoperatively. Surgery for radiculopathy due to spinal metastasis can improve pain in afflicted patients. Postoperative improvement of motor weakness due to spinal metastasis varies depending on the case. Surgery for radiculopathy due to spinal metastasis is indicated when patients have conservative treatment-resistant radiculopathy or amyotrophy that affects their activities of daily living.

## Introduction

The number of cancer survivors has increased worldwide owing to the development of cancer treatment. An increase in survival time leads to an increased incidence of bone metastasis [1]. For patients with radiculopathy due to a metastatic tumor in the spine, nonsurgical treatment is the first choice, as is the case for patients with radiculopathy due to a degenerative spine [2]. We have to consider surgical management of patients who are resistant to conservative treatment, but there are few reports of surgical treatment for radiculopathy due to metastatic tumors in the spine. Moreover, there is no consensus for surgical indications for radiculopathy due to spinal metastasis. We present three cases in patients with radiculopathy due to spinal metastasis who underwent surgery.

## Case presentation

Case 1

**Figure 1:**
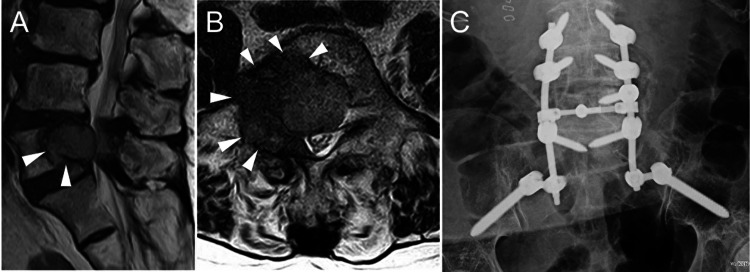
MRI with T2-weighting before surgery and postoperative anteroposterior radiograph (A) Sagittal MRI with T2-weighting revealing a hypointense tumor involving the L5 vertebral body and compressing the thecal sac. (B) Axial MRI with T2-weighting of the lower L5 vertebral body demonstrating a hypointense tumor involving the right L5-S1 foramen. (C) Postoperative anteroposterior radiograph. Pedicle screws were inserted from L3 to S1, and iliac screws were inserted. MRI, magnetic resonance imaging.

An 82-year-old woman underwent surgery 18 years earlier after a diagnosis of breast cancer. She developed right lower limb pain one year earlier, and she had been on conservative treatment. She became unable to walk not because of muscle weakness but because of right leg pain and was referred to our department. MRI revealed a mass in the right L5-S1 intervertebral foramen, an extraforaminal lesion, and right L5 nerve root compression. We diagnosed this as right L5-S1 foraminal stenosis due to breast cancer metastasis to the L5 level of the spine. Her revised Tokuhashi score was 13 points indicating a prognosis of more than one year [3]. After tumor embolization, we performed a subtotal tumor resection and posterior lumbosacral decompression and fusion (Figure 1).

We observed that the right L5 nerve root was severely compressed by the tumor, but the nerve root was decompressed after tumor resection. Intraoperative blood loss was 975 ml. The tissue diagnosis of the tumor was consistent with the earlier carcinoma. Immediately after the operation, the lower limb pain and numbness resolved. After the operation, she was pain-free and was able to walk with the assistance of a walking frame. She also underwent chemotherapy postoperatively. She was moved to palliative care 10 months after surgery and died 11 months after the surgery.

Case 2

**Figure 2:**
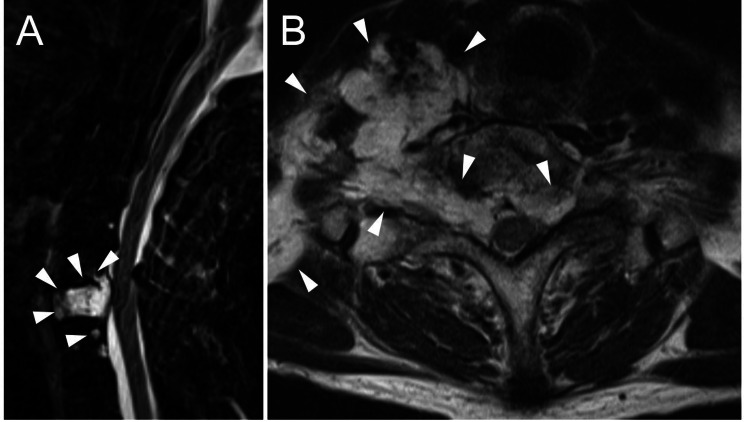
Cervical MRI with a STIR sequence and T2-weighted imaging before surgery (A) Cervical MRI with a STIR sequence showing a hyperintense lesion involving the C7 and T1 (arrowhead) vertebral body and compressing the spinal cord slightly. (B) T2-weighted axial imaging showing a hyperintense epidural tumor (arrowhead) involving both sides of the C7-T1 foramen. The tumor on the right side extends out of the foramen. MRI, magnetic resonance imaging; STIR, short inversion time inversion recovery.

A 70-year-old woman underwent surgery 18 years earlier after a diagnosis of breast cancer and received chemotherapy. She presented with right cervical lymph node metastasis and received radiation therapy 12 years earlier. She was referred to our department because she developed left upper limb pain and muscle weakness of her left finger extension and finger abduction one month earlier. MRI showed an irregular space-occupying lesion extending from the right intervertebral foramen of the C7-T1 to the right brachial plexus and a space-occupying lesion on the left C7-T1 intervertebral foramen on the ventral side (Figure 2).

**Figure 3:**
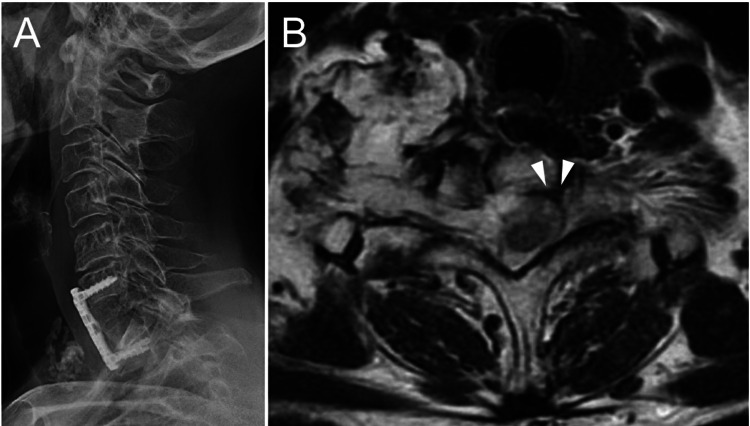
Postoperative lateral radiograph and T2-weighted imaging (A) Postoperative lateral radiograph showing anterior cervical fusion with a plate at the level of C6-T1 with autologous iliac bone grafting. (B) T2-weighted axial imaging revealing complete resection of the tumor in the left C7-T1 foramen (arrowhead) and residual tumor in the right C7-T1 foramen.

She was diagnosed as having left C8 radiculopathy and amyotrophy due to breast cancer metastases to the C7 and T1 vertebrae. Her revised Tokuhashi score was 13 points indicating a prognosis of more than one year [3]. She underwent a corpectomy of C7 and resection of the epidural tumor at the left C7-T1 intervertebral foramen followed by iliac crest autograft and plate fixation from C6 to T1 (Figure 3).

After tumor resection, the emerging C8 nerve root was decompressed. Intraoperative blood loss was only 10 ml. The tissue diagnosis of the tumor was consistent with the earlier carcinoma. She also underwent chemotherapy postoperatively. Although her left upper limb pain improved and the EuroQol 5 Dimension 5 Level (EQ-5D-5L) score [4] increased, muscle weakness of her left finger had not improved at six months after surgery.

Case 3

**Figure 4:**
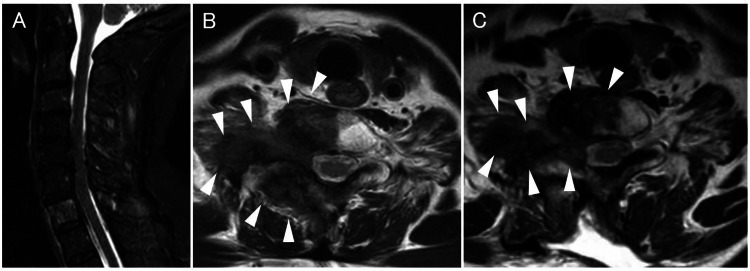
Cervical MRI with a STIR sequence and T2-weighted imaging before and after surgery (A) Cervical MRI with a STIR sequence showing a hyperintense lesion involving the C7 vertebral body and spinous process. (B) T2-weighted axial imaging demonstrating a hypointense space-occupying lesion (arrowhead) involving the right lateral mass of C7 and C7-T1 foraminal space. (C) Postoperative T2-weighted axial imaging showing partial resection of the tumor and residual tumor in right C7-T1 foraminal space (arrowhead). Though postoperative T2-weighted axial imaging is not very different from preoperative T2-weighted axial imaging, her right upper limb pain disappeared and her finger extension muscle strength improved one week after surgery. MRI, magnetic resonance imaging; STIR, short inversion time inversion recovery.

A 72-year-old woman underwent surgery and radiotherapy five years earlier after a diagnosis of rectal cancer. She was referred to our department for pain and numbness in her entire upper right limb. MRI showed a tumor on the right side of the C7 vertebral body and pedicle (Figure 4).

She was diagnosed as having right C8 radiculopathy due to a metastatic tumor in the C7 level of the spine. Although she received conservative treatment with chemotherapy and radiation therapy, three months later, she developed right upper extremity pain, and weakness of her right finger extension, and finger abductor strength progressed. MRI showed mild tumor growth. Her revised Tokuhashi score was 9 points indicating a prognosis of more than six months [3]. Three weeks later, we performed partial tumor resection and left C7-T1 facetectomy. We confirmed that the right C8 nerve root was totally decompressed. Intraoperative blood loss was 50 ml. The tissue diagnosis of the tumor was consistent with the earlier carcinoma. A week after the operation, her right upper limb pain disappeared, and her finger extension muscle strength improved. A month after surgery, she also underwent postoperative chemotherapy. However, two months after the operation, numbness in her right ring finger and little finger recurred, but her muscle strength was maintained, and the EQ-5D-5L [4] score was the same as before surgery. Eight months after the operation, the numbness spread to her right upper arm; however, her muscle strength did not deteriorate. She was diagnosed with meningeal dissemination 11 months after the surgery and moved to palliative treatment. She died 14 months after the operation.

## Discussion

The present cases suggest two important clinical issues. First, surgery for radiculopathy due to spinal metastasis can improve pain in a patient. Second, postoperative improvement of muscle weakness due to spinal metastasis varies depending on the case.

Patients with radiculopathy due to spinal metastasis may benefit from surgery in terms of pain. We often choose nonsurgical treatment for cervical radiculopathy due to degenerative changes because 75%-90% of patients achieve symptomatic improvement with nonoperative care [5,6]. Surgical management is indicated for patients who have persistent pain despite conservative treatment or those who have a significant neurological deficit [5]. It is reasonable to follow the same strategy for cervical radiculopathy due to spinal metastases. When treating metastatic tumors, it is also necessary to consider the patient’s survival expectancy. For the patients with metastatic epidural spinal cord compression (MESCC) or myelopathy due to pathological fracture, surgical treatment is considered when they have an estimated survival of greater than three to six months [7,8]. Whether the criteria for MESCC can be adapted to radiculopathy due to spinal metastasis remains unknown, considering that radiculopathy due to spinal metastasis does not cause serious neurological deficits like paraplegia or bowel bladder dysfunction when compared with MESCC. Thus, surgical treatment for radiculopathy can be justified if the estimated survival is the same or longer than the surgical indication for MESCC. To our knowledge, only six cases of radiculopathy due to spinal metastasis have been reported in the literature, and three were treated by surgery. All patients underwent surgery after they failed nonsurgical treatment. After the surgery, their persistent pain disappeared [9-14]. In the present study, the patients had pain resistant to conservative treatment, and their survival time was estimated as at least six months. In all patients, their pain was relieved, and they could maintain their activities of daily living (ADL) levels for several months.

Postsurgical improvement of muscle weakness due to radiculopathy caused by spinal metastasis varies among cases. In the present three cases, two cases were of metastasis to the cervical spine. Both of the patients showed muscle weakness, and the degree of improvement varied. There were two patients in whom muscle strength was reduced due to radiculopathy caused by spinal metastasis, and surgery was performed as in the previous reports. Surgery was performed after four to six months of conservative treatment. In both cases, although the pain improved, the muscle weakness did not improve well [10,11]. The optimal timing for surgery for cervical radiculopathy due to spinal metastasis accompanying motor weakness is not yet established. In the present case series, the patient with a duration of six weeks from onset to surgery did not recover, whereas patients with a duration of three weeks from onset to surgery had improvement in muscle weakness.

## Conclusions

Surgery for radiculopathy due to spinal metastasis can improve the pain experienced by patients. Postoperative improvement of muscle weakness due to spinal metastasis varies depending on the case. Surgery for patients with radiculopathy due to spinal metastasis is indicated when patients have conservative treatment-resistant radiculopathy or amyotrophy, which affects their ADL. There are few reports about surgery for radiculopathy due to spinal metastasis. Further study is needed to determine indications and the optimal timing for surgery for radiculopathy caused by spinal metastasis accompanying severe pain and motor weakness.
